# Serum Serotonin Levels and Postpartum Depression in Women with Gestational Diabetes Mellitus: A Prospective Psychobiological Study

**DOI:** 10.3390/jcm15145524

**Published:** 2026-07-14

**Authors:** Roba Bdeir, Sarah Al-Ja’freh

**Affiliations:** 1 Department of Basic Scientific Sciences, Al-Balqa Applied University, P.O. Box 206, Al-Salt 19117, Jordan; 2 Department of Midwifery, School of Nursing, Al-Balqa Applied University, P.O. Box 206, Al-Salt 19117, Jordan

**Keywords:** postpartum depression, gestational diabetes mellitus, serotonin, HPA axis, perinatal mental health, EPDS

## Abstract

**Background/Objectives**: Gestational diabetes mellitus (GDM) is an established risk factor for postpartum depression (PPD), yet its psychobiological mechanisms remain poorly characterized. This study aimed to profile the psychological and biochemical-associated factors of PPD among women with GDM in Jordan. **Methods**: A prospective observational study recruited 204 pregnant women from three Jordanian hospitals. The Edinburgh Postnatal Depression Scale (EPDS) was administered antepartum and at 4–9 weeks postpartum, with EPDS ≥ 13 used to define probable PPD. Antepartum serum serotonin, cortisol, and CRP were measured by ELISA in a biomarker subsample of 115 participants (84 non-GDM and 31 GDM). Of 54 women diagnosed with GDM, 44 completed the postpartum EPDS and were included in the within-GDM psychological analysis; 25 of these also had stored antepartum serum and were included in the within-GDM biomarker analysis. Analyses were exploratory, consistent with a pilot design. **Results**: Probable PPD was identified in 26 of 44 GDM women (59.1%). GDM status did not predict PPD in the full cohort (*p* = 0.237). Within the GDM subgroup, women with probable PPD had significantly higher antepartum EPDS scores (*p* = 0.003), and elevated perceived stress (*p* = 0.049), than those without probable PPD. In the within-GDM biomarker subsample (*n* = 25), antepartum serum serotonin was significantly lower in women with probable PPD (*p* < 0.001); CRP and cortisol did not differ significantly. Serotonin and cortisol showed the strongest inverse correlations with postpartum EPDS (both *p* < 0.01). Among GDM women without antepartum depression, 19.0% developed new-onset PPD. **Conclusions**: In our study, GDM women who develop probable PPD exhibit a distinct profile of antenatal vulnerability and lower serum serotonin levels. Routine perinatal mental health screening and biomarker investigation are warranted in this high-risk group. Findings should be confirmed in larger, adequately powered cohorts.

## 1. Key Message

Women with gestational diabetes who developed postpartum depression showed higher antenatal distress and markedly lower serotonin levels. These findings identify a distinct psychobiological vulnerability profile and support integrating mental health screening with biomarker assessment during perinatal care.

## 2. Introduction

Gestational diabetes mellitus (GDM) is a growing public health burden and is rising with obesity rates and sedentary lifestyles [1]. Regionally, a meta-analysis across MENA countries found the average prevalence of GDM was 13.0% [2]. Not surprisingly, the prevalence rate in Jordan was 13.5% [3], as it imposes significant adverse complications on both pregnant women and their babies [4]. Particularly, women with GDM are associated with having higher BMI and higher maternal age, increasing the risk for both mother and baby complications, including gestational hypertension, preeclampsia, and premature birth [5]. If uncontrolled, it also raises risks of neonatal hypoglycemia, jaundice, and stillbirth [6].

A growing body of evidence, including several large meta-analyses, has identified GDM as a probable risk factor for postpartum depression (PPD), although effect estimates vary considerably across populations and study designs [7,8,9]. The most recent, published in 2025, evaluated 29 studies involving 2,442,001 participants and reported that women with GDM had a 42% increased risk of developing PPD (RR = 1.42, 95% CI: 1.17–1.72) [7]. Similarly, S. Hinkle and colleagues (2016), in a prospective cohort study of 2477 pregnant women followed up at 6 weeks postpartum, observed an even stronger association, reporting that GDM is associated with a 4.62-fold increased risk of PPD (adjusted RR = 4.62, 95% CI: 1.26–16.98) [9].

PPD affects 10–20% of women globally [10], with substantially higher rates in the Middle East and Jordan [11]. In Jordan specifically, prevalence among the general Jordanian population reaches 22% [12]. PPD causes significant maternal suffering, contributing to chronic depression, suicidal ideation, and reduced quality of life [13], and critically, it impairs child development through disrupted mother–infant bonding [14].

GDM and depression share an intersecting biological pathway, where insulin resistance impairs tryptophan transport across the blood–brain barrier, reducing central serotonin synthesis [15,16]. Moreover, chronic low-grade inflammation has been proposed as one candidate mechanism linking GDM to postpartum depressive symptoms, with elevated C-reactive protein (CRP) and pro-inflammatory cytokines reported in both conditions. However, perinatal studies of CRP and depressive symptoms have yielded heterogeneous results, including null and inverse associations, and the role of systemic inflammation in PPD remains contested [17,18,19]. A more recent mechanistic model further proposed that insulin resistance disrupts glucose metabolism in the central nervous system, affecting dopamine and serotonin signaling pathways [20]. Nevertheless, despite these proposed mechanisms, there are still limited studies thus far directly linking tryptophan transport and serotonergic dysfunction in relation to PPD within the GDM population. In particular, there is a lack of large-scale human clinical studies investigating these pathways. Most existing studies are cross-sectional in design, and they lack biomarker assessment [21,22,23]. To date, no study in the Arab world has simultaneously investigated serotonin, cortisol, and C-reactive protein (CRP) as variables associated with PPD specifically among women with GDM.

Therefore, this prospective study aimed to characterize the psychobiological profile of PPD among women with GDM in Jordan, with a specific focus on the role of serum serotonin, cortisol, and CRP as biomarkers of PPD risk within this high-risk metabolic subgroup.

## 3. Materials and Methods

### 3.1. Study Design and Participants

This study employed a prospective observational design to investigate psychological, psychosocial, and biochemical characteristics associated with postpartum depression (PPD) among pregnant women with gestational diabetes mellitus (GDM). A total of 204 pregnant women were recruited during the third trimester of pregnancy from multiple antenatal and postpartum care services from three different hospitals across north and central Jordan. Participants were categorized into two groups according to GDM status: women diagnosed with GDM and non-GDM controls.

**Figure 1:**
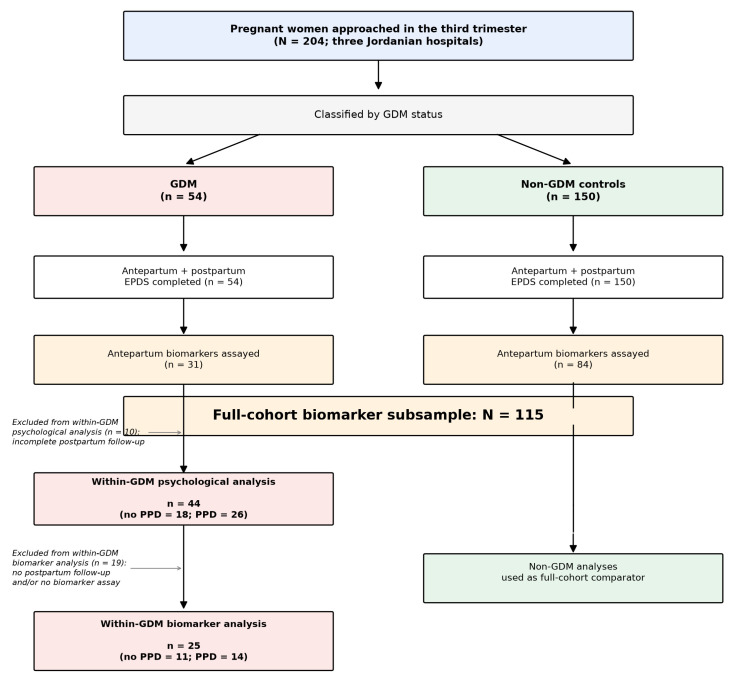
Participant flow diagram and the analytic samples used for each comparison. Flow of participants through recruitment, gestational diabetes classification, psychological follow-up, and biomarker analyses. Reasons for attrition at each step (incomplete postpartum follow-up, insufficient stored serum/budget-limited assay capacity, pre-existing psychiatric diagnosis).

Pregnant women aged 18 years and older were eligible to participate. Participants were recruited during the third trimester of their pregnancy and followed into the postpartum period. Women were included if they were pregnant during the recruitment period and agreed to participate voluntarily. Participants with pre-existing psychiatric disorders diagnosed prior to pregnancy or incomplete clinical or biochemical data were excluded from the final analysis. The participant flow through recruitment, follow-up, and biomarker analysis is summarized in Figure 1.

### 3.2. Diagnosis of Gestational Diabetes Mellitus

Gestational diabetes mellitus was diagnosed according to the International Association of Diabetes and Pregnancy Study Groups (IADPSG) criteria using a one-step 75 g oral glucose tolerance test (OGTT) performed at 24–28 weeks of gestation. A diagnosis of GDM was established when one or more of the following plasma glucose thresholds were met or exceeded: fasting ≥ 5.1 mmol/L (92 mg/dL), 1 h ≥ 10.0 mmol/L (180 mg/dL), or 2 h ≥ 8.5 mmol/L (153 mg/dL). The diagnosis was confirmed by the treating obstetrician and verified from the participant’s hospital antenatal records. Women with pre-gestational type 1 or type 2 diabetes were not eligible.

### 3.3. Clinical and Demographic Data Collection

Sociodemographic, lifestyle, psychosocial, and obstetric data were collected using structured interviewer-administered questionnaires and medical record review. Variables collected included maternal age, body mass index (BMI), educational status, household income, smoking exposure, exercise habits, relationship status, debt, trauma exposure, parity, gestational age, prior pregnancy complications, delivery mode, and pregnancy risk classification. Participants were classified according to BMI categories based on World Health Organization (WHO) criteria. High-risk pregnancy status was determined according to obstetric evaluation documented in clinical records.

### 3.4. Assessment of Depressive Symptoms and Social Support

Antenatal and postpartum depressive symptoms were assessed using the Edinburgh Postnatal Depression Scale (EPDS) during the third trimester of pregnancy (≥26 weeks’ gestation) and again during the early postpartum period. The postpartum assessment window ranged from 4 to 9 weeks (median [IQR]: 6.4 [4] weeks). The EPDS is a validated questionnaire with 10 self-report instruments; each item is scored 0–3, with a total score ranging from 0 to 30. The validated Arabic version of the EPDS, previously adapted and validated in various Middle East perinatal populations [24,25,26], was administered. Because the EPDS is a screening instrument rather than a diagnostic interview, the outcome is described throughout as “probable postpartum depression” rather than a confirmed clinical diagnosis of postpartum depression. Probable postpartum depression was defined as an EPDS total score of ≥13, the threshold most commonly applied in Arabic-language validation studies; this cut-off has been used consistently in all tables, figures, and text in the revised manuscript.

Any non-zero response on EPDS item 10 (thoughts of self-harm) was reviewed by the research team and communicated to the participant’s treating obstetrician for clinical follow-up and onward referral to mental health services as deemed clinically appropriate. Participants who screened in the probable-depression range (EPDS ≥ 13) were also informed of their score and advised to seek follow-up with their treating clinician. This safeguarding pathway was consistent with the institutional ethics committee approval and the participant consent procedure.

Perceived social support was assessed using the Multidimensional Scale of Perceived Social Support (MSPSS), a 12-item self-report instrument originally developed by Zimet et al. (1988) [27], administered in the Arabic version previously validated in various Middle Eastern adult populations [28,29,30] evaluating support from family, friends, and the significant other (husband) using a 5-point Likert scale, yielding a total score from 10 to 60. Total MSPSS scores were categorized as low (≤36), moderate (37–48), or high (49–60) perceived social support. The MSPSS was administered at the postpartum visit (4–9 weeks postpartum) together with the postpartum EPDS. Higher scores indicate greater perceived social support. Because perceived social support and postpartum depressive symptoms were measured during the same postpartum visit, the cross-sectional association between them is reported as exploratory, and the direction of effect cannot be inferred.

### 3.5. Blood Sampling and Biochemical Analysis

Peripheral venous blood samples were collected into plain tubes from participants during their third trimester of pregnancy at the antenatal clinic visit, in the morning (between approximately 08:00 and 10:00) after an overnight fast, to minimize diurnal and prandial variation in serum cortisol and serotonin. Participants with acute infection, active inflammatory conditions, or current use of medications known to affect serotonergic or HPA-axis function (including selective serotonin reuptake inhibitors, other antidepressants, systemic corticosteroids, and anti-inflammatory agents at the time of sampling) were excluded at recruitment. The blood tube was centrifuged at 3000 rpm for 10 min at room temperature within 30 min of collection. Serum was subsequently separated and stored at −80 °C and underwent a single freeze–thaw cycle prior to analysis. Serum serotonin (5-hydroxytryptamine), cortisol, and high-sensitivity C-reactive protein (CRP) concentrations were measured using commercial enzyme-linked immunosorbent assay (ELISA) kits by Genochem World S.L. (Valencia, Spain). The catalog numbers for the kits were as follows: serotonin (GW0310Hu), cortisol (GW0307Hu), and CRP (GW0309Hu). Because of resource constraints, samples were assayed once rather than in duplicate, and kit-specific intra- and inter-assay coefficients of variation and lower limits of detection were not independently re-verified in our laboratory; the manufacturers’ reported assay performance characteristics were used. Biomarker concentrations demonstrated non-normal distributions and were log-transformed prior to inferential statistical analyses. Serum serotonin measurements were interpreted cautiously, as measured concentrations may partially reflect platelet-derived serotonin release during sample processing in addition to free circulating serotonin.

### 3.6. Statistical Analysis

Statistical analyses were performed using IBM SPSS Statistics version 26. Continuous variables were assessed for normality using the Shapiro–Wilk test and visual inspection of Q–Q plots. Normally distributed continuous variables are presented as mean ± standard deviation (SD); categorical variables are presented as frequencies and percentages. Serum biomarker concentrations were log-transformed prior to inferential analysis after confirmation of approximate normality on the transformed scale.

Three nested analytic samples were used in this study, as detailed in Figure 1 (participant flow diagram):

1. Full cohort (N = 204; non-GDM *n* = 150, GDM *n* = 54)—used for demographic, obstetric, and psychological comparisons by GDM status (Table 1; psychological/psychosocial rows of Table 2).
2. Full-cohort biomarker subsample (N = 115; non-GDM *n* = 84, GDM *n* = 31)—used for between-group antepartum biomarker comparisons (biomarker rows of Table 2). The reduction from 204 to 115 reflects budget-limited assay capacity.
3. Within-GDM analytic samples—*n* = 44 GDM women with complete postpartum follow-up were included in the within-GDM psychological analysis (Table 3, psychological section; no PPD = 18, PPD = 26); *n* = 25 of these also had stored antepartum serum and were included in the within-GDM biomarker analysis (Table 3, biomarker section; no PPD = 11, PPD = 14).

The analytic sample applied to each comparison is stated explicitly in every table and figure legend. Comparisons between groups were performed using independent-samples *t*-tests for continuous variables and chi-square tests for categorical variables, with Welch’s correction applied where equality-of-variances assumptions were violated. Spearman’s rank correlation analysis evaluated associations between depressive symptoms, psychosocial measures, and biochemical markers within the GDM subgroup. Given the small biomarker subsample and the exploratory nature of these analyses, biomarker findings should be interpreted as hypothesis-generating. A two-tailed *p*-value < 0.05 was considered statistically significant.

## 4. Results

### 4.1. Participant Flow and Analytic Samples

A total of 204 pregnant women were screened and enrolled in the third trimester; 54 were diagnosed with GDM, and 150 were classified as non-GDM. Of the 54 women with GDM, 44 completed the postpartum EPDS assessment at 4–9 weeks and formed the within-GDM psychological analysis sample (Table 3), and 25 of these also had complete third-trimester biomarker data and formed the within-GDM biomarker subsample (Table 3). Serum serotonin, cortisol, and CRP were available for 115 participants overall (non-GDM *n* = 84; GDM *n* = 31) and constituted the full-cohort biomarker subsample (Table 2). The full participant flow, including reasons for exclusion and loss to follow-up, is shown in Figure 1.

### 4.2. Participant Characteristics

A total of 204 women were included in the analysis, of whom 150 (73.5%) had no GDM and served as controls, and 54 (26.5%) were diagnosed with GDM. Table 1 summarizes the demographic, psychosocial, and obstetric characteristics stratified by GDM status. Women with GDM were significantly older than non-GDM participants (34.74 ± 5.73 vs. 29.67 ± 5.88 years, *p* = 0.002). Advanced maternal age, women aged more than 35 years, was significantly more common among women with GDM (50.0% vs. 24.7%, *p* < 0.001). Women with GDM were also associated with significantly higher BMI values (32.93 ± 4.83 vs. 30.21 ± 5.93 kg/m^2^, *p* = 0.002), with obesity being substantially more prevalent among the GDM group (74.1% vs. 50.0%, *p* = 0.002). Regarding psychosocial characteristics, recent trauma exposure was significantly more prevalent among women with GDM compared with non-GDM participants (57.4% vs. 36%, *p* = 0.008). No significant differences were observed between groups with respect to exercise, household income, smoking status, smoke exposure, relationship status, debt, or perceived stress scores.

Obstetrically, women with GDM were recruited at a slightly earlier gestational age (33.01 ± 3.65 vs. 34.78 ± 3.69 weeks, *p* = 0.003). Prior pregnancy complications were significantly more common among women with GDM (77.8% vs. 44%, *p* < 0.001). Women with GDM also delivered at an earlier gestational age compared with non-GDM participants (36.57 ± 1.89 vs. 38.41 ± 1.77 weeks, *p <* 0.001). No significant differences were observed regarding parity, cesarean delivery rates, or preterm birth frequency.

### 4.3. Depression and Social Support Profiles

Of the 204 recruited participants, all completed both antenatal and postpartum assessments and were included in the PPD outcome analyses. Demographic and depression outcome analyses by GDM status are reported for the full cohort (N = 204), while biomarker analyses are restricted to 115 participants (N = 115).

Antepartum and postpartum for both EPDS scores and social support scores stratified by GDM status are presented in Table 2. Antepartum EPDS scores did not significantly differ between women with and without GDM. Similarly, no significant differences were observed in the prevalence of probable antepartum or postpartum depression between groups. Antepartum social support scores did not significantly differ between groups. However, postpartum EPDS scores (13.59 ± 6.27 vs. 10.91 ± 6.99, *p* = 0.014) and postpartum social support scores (50.86 ± 8.26 vs. 47.88 ± 8.11, *p* = 0.038) were significantly higher among women with GDM compared with non-GDM participants. Notably, among women with GDM who did not exhibit antepartum depressive symptoms (EPDS < 13, *n* = 21), 4 subsequently developed postpartum depressive symptoms, representing a new-onset PPD rate of 19.0% within this subgroup.

### 4.4. Biochemical Levels According to GDM Status

Antepartum biomarker serum levels stratified by GDM status are summarized in Table 2. No statistically significant differences were observed between women with and without GDM regarding serum CRP, cortisol, or serotonin levels.

### 4.5. Psychological and Biomarker Characteristics Within GDM Women

Among women with GDM, Table 3 shows characteristics of GDM women stratified by postpartum depression status. Those who developed probable PPD displayed significantly higher antepartum EPDS scores (16.62 ± 5.21 vs. 11.17 ± 6.40, *p* = 0.003). Probable antepartum depression was also substantially more prevalent among women who later developed postpartum depression (84.6% vs. 38.9%, *p* = 0.002). Women with postpartum depression were associated with significantly higher stress scores (2.31 ± 1.26 vs. 1.67 ± 0.84, *p* = 0.049). The median postpartum assessment week did not differ significantly between women with and without PPD 6.5 vs. 6.3 weeks, *p* = 0.42), and inclusion of postpartum week as a covariate did not materially alter the observed associations.

Antepartum social support scores did not significantly differ between groups; however, postpartum social support scores were significantly higher among women with probable PPD (53.77 ± 7.45 vs. 46.67 ± 7.71, *p* = 0.004). Higher postpartum social support scores among women with probable PPD may reflect reactive mobilization of social support following the emergence of depressive symptoms rather than a protective effect.

**Figure 2:**
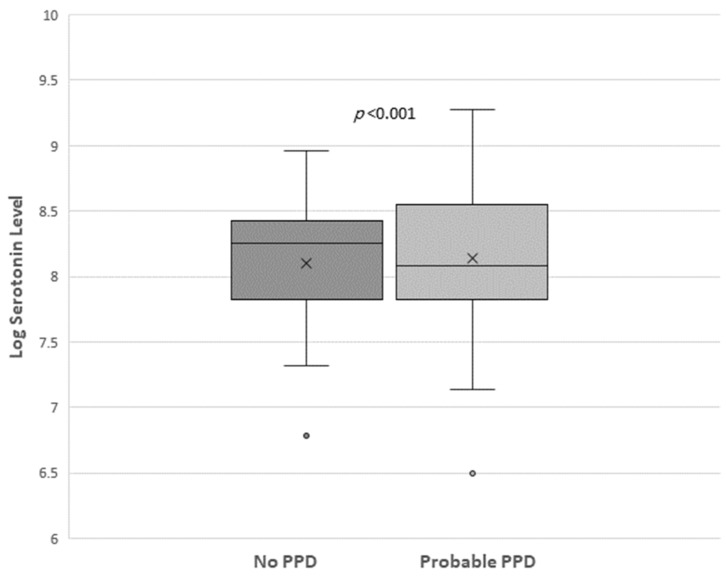
Log-transformed serotonin levels among women with GDM according to PPD status. Women with GDM and probable postpartum depression (EPDS ≥ 13, *n* = 14) were associated with significantly lower log-transformed serotonin levels, *p* < 0.001, compared with women with GDM without postpartum depression (*n* = 11). Boxes represent the interquartile range, horizontal lines represent the median, and whiskers represent the range of observations, while points represent outliers.

Biomarker analysis revealed significantly lower serotonin levels among women with postpartum depression compared with women without postpartum depression (7.55 ± 0.44 vs. 8.33 ± 0.44, *p* < 0.001). Women with probable postpartum depression were associated with lower serotonin levels compared with women without postpartum depression. As illustrated in Figure 2, the distribution of log-transformed serum serotonin levels among women with GDM according to postpartum depression status was significantly lower. The median serotonin level was visibly lower in the PPD group, underscoring the strength of this association. In contrast, cortisol levels did not significantly differ between groups. Antepartum log-transformed CRP did not differ significantly between women with and without GDM (full-cohort biomarker subsample, *n* = 115; Table 2), nor between women with and without probable PPD within the GDM subgroup (within-GDM biomarker subsample, *n* = 25; Table 3). In exploratory correlation analyses, however, log-CRP showed a weak-to-moderate inverse association with both antepartum and postpartum EPDS scores (Table 4: r = −0.433, *p* < 0.05). These two findings describe different analyses, between-group comparison of mean CRP versus continuous correlation with EPDS, and are reported as exploratory.

### 4.6. Correlation Analysis

**Figure 3:**
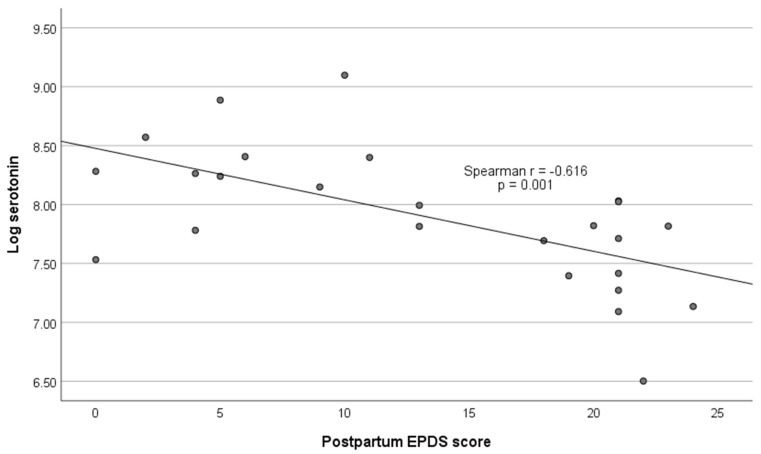
Inverse association between postpartum EPDS score and serotonin levels within the women with GDM subgroup.

Spearman’s correlation analysis, summarized in Table 4, was associated with significant positive correlations between stress scores and both antepartum EPDS scores (r = 0.494, *p* < 0.01) and postpartum EPDS scores (r = 0.351, *p* < 0.05). Antepartum EPDS scores were positively associated with postpartum EPDS scores (r = 0.481, *p* < 0.01), suggesting continuity of depressive symptoms across the perinatal period. Postpartum EPDS scores were associated with significant inverse correlations with both log-transformed serum cortisol (r = −0.626, *p* < 0.01) and serotonin levels (r = −0.616, *p* < 0.01), indicating that lower levels of both were associated with greater postpartum depressive symptom severity. As illustrated in Figure 3, higher postpartum EPDS scores were associated with lower serotonin levels (Spearman’s r = −0.616, *p* = 0.001).

Additionally, postpartum social support scores were associated with a significant inverse correlation with serotonin levels (r = −0.452, *p* < 0.05). This unexpected finding may reflect reactive increases in family or social involvement following the emergence of depressive symptoms. CRP levels demonstrated significant inverse correlations with both antepartum EPDS (r = −0.433, *p* < 0.05) and postpartum EPDS scores (r = −0.473, *p* < 0.05).

## 5. Discussion

In the present study, PPD was common among women with GDM, with 59.1% of women with GDM demonstrating probable PPD based on EPDS scores. Although GDM status itself was not independently associated with increased PPD risk in the full cohort (*p* = 0.237), analyses within the GDM subgroup revealed a distinct psychobiological profile that clearly differentiated them from those who did not develop PPD (*p* < 0.001). More specifically, women with GDM who had probable PPD had higher antepartum depressive symptoms, elevated stress scores, and altered serotonergic signaling. The observation that women with probable PPD reported higher postpartum perceived social support than non-depressed women is counterintuitive and should be interpreted with caution. Postpartum support and depressive symptoms were measured concurrently, so reverse causality is plausible: emergent depressive symptoms may mobilize family and partner support, particularly in Jordanian and broader Arab cultural contexts where extended-family involvement after childbirth is normative.

Contrary to the inflammation-based model linking GDM to postpartum depressive symptoms, CRP did not differ between women with and without GDM, nor between women with and without probable PPD, and was inversely, not positively, correlated with antepartum and postpartum EPDS scores. Several interpretations are possible. First, a single antepartum high-sensitivity CRP measurement is a coarse index of chronic low-grade inflammation and may be influenced by acute physiological variation despite the exclusion of women with overt infection at sampling. Second, the inverse association may reflect chance in a small biomarker subsample, residual confounding by BMI or gestational age, or reverse pathways in which depressive symptoms attenuate acute-phase reactants. Third, the finding is not unprecedented: several perinatal studies have reported null or inverse CRP–depressive symptom associations, suggesting that the relationship between systemic inflammation and PPD is more heterogeneous than the cytokine-based hypothesis would predict. The CRP findings should, therefore, be regarded as exploratory and as inconsistent with a simple inflammation model in this cohort.

Most notably, peripheral serotonin emerged as the largest between-group difference in this exploratory sample of PPD symptom severity within the GDM subgroup. Specifically, GDM women who developed PPD had significantly lower serum serotonin levels compared with those who did not develop PPD (7.55 ± 0.44 vs. 8.33 ± 0.44 log-transformed units, *p* < 0.001), with a strong inverse correlation between serotonin and postpartum EPDS scores (r = −0.616, *p* < 0.01). In contrast, inflammatory and neuroendocrine markers, CRP and cortisol, did not significantly differ between GDM women with and without PPD.

Although prior meta-analyses report an elevated PPD risk associated with GDM (7–9), GDM status was not an independent predictor of PPD in our full cohort (*p* = 0.237). Several factors likely contribute to this apparent discrepancy. This study was not powered to detect the modest pooled effect reported in the most recent meta-analysis (RR ≈ 1.42) (7); with only 54 GDM cases, the confidence interval around our group comparison is wide and compatible with effects of that magnitude. The non-GDM comparison group in this Jordanian sample also exhibited a high baseline PPD prevalence, consistent with regional estimates of 22% (12), which would attenuate any between-group difference. Population-specific psychosocial exposures, including the high prevalence of recent trauma observed here, may further blunt the incremental contribution of GDM. Importantly, the absence of a between-group difference at the cohort level does not negate the within-GDM findings: among women with GDM, a distinct antenatal and serotonergic profile clearly differentiated those who went on to develop PPD.

Within the metabolic context of GDM, insulin resistance, a defining feature of GDM, has been found to impair tryptophan transport and availability across the blood–brain barrier, thus reducing central serotonin synthesis and altering the mood regulation pathway in the brain [16,20]. During the postpartum period, rapid estrogen withdrawal leads to further suppression of serotonergic activity by downregulating tryptophan hydroxylase [31]. In women with GDM, these two mechanisms, impairment of serotonin precursor availability and postpartum hormonal shifts, come together and may create a low serotonin state that increases vulnerability and predisposition to PPD.

Nevertheless, serum serotonin measurements should be interpreted cautiously, as serum serotonin levels primarily reflect two sources: platelet-derived serotonin released during the coagulation process alongside the serotonin synthesized from enterochromaffin cells in the gastrointestinal tract, which is rapidly taken up by the platelets via the serotonin transporter (SERT) [32]. This differs from the direct central nervous system serotonin availability, as it does not readily cross the blood–brain barrier; thus, serotonin pools are compartmentally separated and tissue-specific [33]. In GDM specifically, lower serum serotonin levels appear to be tissue-specific as well; Li and colleagues demonstrated downregulation of placental trophoblast serotonin uptake due to impaired SERT translocation to the cell surface [34], despite the unaffected circulating serotonin levels in the blood. This finding indicates that peripheral serotonin levels do not accurately reflect the tissue-specific availability in GDM. Similarly, a study that compared GDM placental tissue with non-GDM pregnancies found a significantly reduced expression of SERT, at both mRNA and protein levels, exhibiting altered placental serotonergic signaling and lower serum serotonin levels [35].

Critically, however, the SERT dysfunction in the placenta described by Li and colleagues was not observed in SERT within platelets, where function was preserved [34]. Since serotonin in our study was measured from serum, which primarily reflects serotonin stored in platelets rather than placental tissue, placental SERT dysfunction described in the experimental GDM models thus far is unlikely to directly explain our findings. Rather, the low serum serotonin observed in GDM women with PPD more plausibly reflects reduced platelet serotonin content or uptake. This is more consistent with established evidence linking low platelet.

However, Li et al. found preserved SERT function in platelets, indicating that placental SERT dysfunction may not directly affect serum serotonin measurements, which primarily reflect platelet serotonin [34]. Therefore, the lower serum serotonin observed in GDM women with PPD is more likely to reflect reduced platelet serotonin content or uptake, consistent with well-established evidence linking low platelet serotonin to PPD. Particularly, untreated PPD patients were shown to have a 50% reduction in platelet serotonin levels [36] and altered platelet serotonin transporter binding within women exhibiting PPD [37]. Moreover, a notable finding was that 19% of the GDM women who had no antepartum depressive symptoms ultimately developed new-onset PPD. Their PPD is less likely to be explained by pre-existing psychological vulnerability, further enforcing that biological mechanisms may play a more critical role. The observed variations in serotonin levels, therefore, represent a potential biomarker that should be further investigated in larger prospective studies. In addition, serum serotonin levels alone cannot confirm central serotonergic deficiency. Future GDM studies should also evaluate tryptophan metabolism markers, including the kynurenine-to-tryptophan ratio, inflammatory markers, and platelet SERT expression and activity to better understand the neurobiological mechanisms linking GDM and PPD.

During pregnancy, the placenta produces large amounts of corticotropin-releasing hormone (CRH), which is stimulated by the hypothalamic–pituitary–adrenal (HPA) axis, resulting in higher circulating cortisol levels. Postpartum results in an abrupt withdrawal of placental CRH and a dramatic decrease in cortisol levels due to HPA axis hyporesponsiveness. It has been proposed that PPD may involve an impaired or blunted stress-response system following childbirth, as women who fail to recover adequate HPA axis reactivity may be particularly vulnerable to PPD. Similar to our observation, where a strong inverse relationship between cortisol levels and PPD scores was found, multiple studies demonstrated lower cortisol levels associated with greater depressive severity, supporting blunting [6,38,39,40]. A systematic review of 48 studies found that the majority identified HPA abnormalities and consistently pointed toward an attenuated response in women with PPD [41]. Nonetheless, the literature reveals mixed evidence for hyperactivation, hypoactivation, and complex dysregulation patterns of HPA axis abnormalities in PPD [42,43,44]. This discrepancy may reflect differences in sample method and measurement timing, antepartum stage, and population/individuals characteristics. Additionally, variability in the timing of PPD assessment, which ranged from 4–12 weeks postpartum, may also be a contributing factor to the attenuation of group differences. Subsequently, these findings warrant replication in larger GDM cohorts with more standardization of PPD assessment tools and variability, and using longitudinal cortisol sampling to further understand the HPA-axis dysregulation and depressive symptoms over time during pregnancy and after delivery. Cortisol was measured on a single fasting morning antepartum sample. Although an inverse correlation with postpartum EPDS was observed in the within-GDM biomarker subsample, a single antepartum value cannot establish postpartum HPA-axis hyporesponsiveness. This finding is reported as exploratory; longitudinal peripartum sampling and diurnal profiling would be required to support any HPA-axis interpretation.

Psychosocial factors during pregnancy also appeared to contribute to PPD vulnerability among women with GDM, where both antepartum depressive symptoms and stress scores were significantly higher. GDM diagnosis and management itself can be an emotional burden on these women, considering the strict diet, repeated glucose monitoring, and fear of abortion or complications [45]. Also, GDM has been associated with a high prevalence of negative emotions, including worry, disappointment, upset, sadness, and anxiety during the antepartum and postpartum period [46]. This is consistent with previous literature suggesting antenatal depressive symptoms are one of the strongest predictors of PPD [47,48,49].

This study has several strengths; to our knowledge, it is among the few studies in the Arab region to prospectively evaluate psychological, psychosocial, and biochemical markers of PPD specifically among women with GDM. Nevertheless, several limitations must be addressed. First, the sample size is modest: of 204 women recruited, 54 were diagnosed with GDM, 44 completed postpartum follow-up and contributed to the within-GDM subgroup analysis, and biomarkers were quantified in only 32. This restricts statistical power, precludes multivariable adjustment for potential confounders, and limits the generalizability of subgroup estimates. Accordingly, we frame this work as a pilot study, intended to characterize a candidate psychobiological profile that warrants confirmation in adequately powered cohorts. Due to limited budgeting, the biomarkers were not measured across the entire cohort population but rather in a subgroup, limiting statistical power and generalizability. CRP was assayed only once antepartum, and no cytokine panel (e.g., IL-6, TNF-α) was measured, precluding strong inference about systemic inflammation as a mechanism linking GDM and PPD in this cohort. Additionally, we were not able to extract records on fasting glucose and HbA1c levels for these patients, and they were not assessed alongside biomarker analysis. Furthermore, the absence of information on GDM treatment, the use of a single antenatal biomarker measurement, and the lack of postpartum biomarker reassessment are acknowledged limitations of the current study. Finally, the postpartum assessment window of 4–9 weeks is relatively wide; emotional state and neuroendocrine parameters can shift substantially across this interval, and although follow-up timing did not significantly differ between PPD and non-PPD groups (see below), residual timing-related variability cannot be excluded.

## 6. Conclusions

In conclusion, this prospective study suggests that women with GDM who develop PPD constitute a psychologically and biologically distinct subgroup, characterized by greater antenatal distress and lower peripheral serotonin levels. Given the limited sample size and exploratory nature of the biomarker analyses, these findings should be regarded as preliminary and hypothesis-generating rather than definitive. They nonetheless support the value of routine perinatal mental health screening using the EPDS in women with GDM and motivate adequately powered longitudinal studies integrating serotonin, HPA-axis, and tryptophan-metabolism markers to clarify the mechanisms linking GDM and PPD.

## Figures and Tables

**Table 1:** Demographic, psychosocial, and obstetric characteristics of participants according to GDM status of the full cohort (N = 204; GDM *n* = 54, non-GDM *n* = 150).

	Variable	No GDM (*n* = 150)	GDM (*n* = 54)	*p*-Value
**Demographics**	**Age (years)**	30.58 ± 5.42	34.74 ± 5.73	**<0.001**
	**Age group**			**<0.001**
	Middle maternal age (ref) (25–34)	93 (62.0)	25 (46.3)	
	Young maternal age (<25)	20 (13.3)	2 (3.7)	
	Advanced maternal age (≥35)	37 (24.7)	27 (50.0)	
	**BMI**	29.67 ± 5.88	32.93 ± 4.83	**0.002**
	**BMI group**			**0.002**
	Normal weight (ref) (18.5–24.9)	25 (16.7)	1 (1.9)	
	Overweight (25.0–29.9)	50 (33.3)	13 (24.1)	
	Obese (>30)	75 (50.0)	40 (74.1)	
	**Educational status**			0.742
	Bachelor (ref)	56 (37.3)	19 (35.2)	
	Uneducated	23 (15.3)	6 (11.1)	
	High school/Diploma	55 (36.7)	24 (44.4)	
	Post-graduate	16 (10.7)	5 (9.3)	
	**Residency**			0.501
	Urban (ref)	98 (65.3)	38 (70.4)	
	Rural	52 (34.7)	16 (29.6)	
	**Income group**			0.117
	Middle 350–600 JD (ref)	84 (56.0)	24 (44.4)	
	Low < 350 JD	45 (30.0)	16 (29.6)	
	High > 600 JD	21 (14.0)	14 (25.9)	
**Lifestyle/Psychosocial**	**Exercise**			0.512
	No activity	122 (81.9)	42 (77.8)	
	Any activity	27 (18.1)	12 (22.2)	
	**Mother smoking status**			0.947
	Non-smoker	116 (77.3)	42 (77.8)	
	Ever-smoker	34 (22.7)	12 (22.2)	
	**Smoke Exposure**			0.995
	No exposure	27 (18.0)	10 (18.5)	
	Second hand only	89 (59.3)	32 (59.3)	
	Maternal active smoking	34 (22.7)	12 (22.2)	
	**Relationship status**			0.460
	Good	121 (80.7)	41 (75.9)	
	Average/Poor	29 (19.3)	13 (24.1)	
	**Recent trauma event**			**0.006**
	No	96 (64.0)	23 (42.6)	
	Yes	54 (36.0)	31 (57.4)	
	**Debt**			0.755
	No	63 (42.0)	24 (44.4)	
	Yes	87 (58.0)	30 (55.6)	
	**Stress score**	1.71 ± 1.07	1.98 ± 1.07	0.140
**Obstetric**	**Gestational age at recruitment (weeks)**	34.78 ± 3.69	33.01 ± 3.65	**0.003**
	**Parity Group**			0.296
	Primigravida (first child)	38 (25.3)	10 (18.5)	
	1–2 children	65 (43.3)	21 (38.9)	
	3+ children (grandmultipara)	47 (31.3)	23 (42.6)	
	**Prior complications with previous pregnancies**	66 (44.0)	42 (77.8)	**<0.001**
	**Cesarean Delivery**	68 (53.1)	27 (61.4)	0.343
	**Gestational age at delivery (weeks)**	38.41 ± 1.77	36.57 ± 1.89	**<0.001**
	**Preterm Delivery (<37 weeks)**	17 (13.3)	11 (25.0)	0.069

Continuous normal variables were reported as mean ± SD and compared using an independent *t*-test; categorical variables were reported as *n* (%) using a chi-square test. *p*-values < 0.05 were considered significant and presented in bold.

**Table 2:** Antepartum and postpartum EPDS, social support scores, and biomarker levels of participants according to GDM status.

Variable	No GDM	GDM	*p*-Value
**Psychological and psychosocial measures** (full cohort, N = 204; non-GDM *n* = 150, GDM *n* = 54)			
**EPDS total score (antepartum)**	10.91 ± 6.99	13.59 ± 6.27	**0.014**
**EPDS level (antepartum)**			0.057
No antepartum depression indicated (score < 13)	81 (54.0)	21 (38.9)	
Probable antepartum depression (score ≥ 13)	69 (46.0)	33 (61.1)	
**Antepartum social support (total score)**	46.55 ± 7.77	47.37 ± 7.78	0.505
**Social support score level (antepartum)**			0.828
Low support (score < 36)	15 (10.0)	4 (7.4)	
Moderate support (score 37–48)	54 (36.0)	21 (38.9)	
High support (score 49–60)	81 (54.0)	29 (53.7)	
**EPDS total score (Postpartum)**	11.63 ± 6.79	13.66 ± 8.44	0.154
**EPDS level (Postpartum)**			0.086
No postpartum depression indicated (score < 13)	71 (55.9)	18 (40.9)	
Probable postpartum depression (score ≥ 13)	56 (44.1)	26 (59.1)	
**Postpartum social support (total score)**	47.88 ± 8.11	50.86 ± 8.26	**0.038**
**Social support score level (postpartum)**			0.353
Low support (score < 36)	9 (7.0)	2 (4.5)	
Moderate support (score 37–48)	65 (50.8)	18 (40.9)	
High support (score 49–60)	54 (42.2)	24 (54.5)	
**Antepartum serum biomarkers** (biomarker subsample, N = 115; non-GDM *n* = 84, GDM *n* = 31)			
**log CRP (mg/L)**	1.38 ± 0.27	1.38 ± 0.31	0.993
**log Cortisol (µg/dL)**	3.82 ± 0.83	3.58 ± 0.98	0.172
**log Serotonin (ng/mL)**	7.81 ± 0.71	7.88 ± 0.61	0.648

Continuous normal variables were reported as mean ± SD and compared using an independent *t*-test; categorical variables were reported as *n* (%) using a chi-square test. *p*-values < 0.05 were considered significant and presented in bold. Biomarker variables were log-transformed prior to inferential analyses due to non-normal distribution. The psychological/psychosocial section is based on the full cohort; the biomarker section is restricted to the subsample with available antepartum assays (N = 115).

**Table 3:** Psychological, psychosocial, and biomarker characteristics among women with GDM according to PPD status.

Variable	No PPD	PPD	*p*-Value
**Psychological and psychosocial characteristics** (within-GDM analytic sample, *n* = 44; no PPD 18, PPD 26)			
**Antepartum EPDS score**	11.17 ± 6.40	16.62 ± 5.21	**0.003**
**Antepartum probable depression**	7 (38.9)	22 (84.6)	**0.002**
**Antepartum social support total**	48.56 ± 6.58	45.73 ± 7.53	0.205
**Postpartum social support total**	46.67 ± 7.71	53.77 ± 7.45	**0.004**
**Stress score**	1.67 ± 0.84	2.31 ± 1.26	**0.049**
**Antepartum serum biomarkers** (within-GDM biomarker sample, *n* = 25; no PPD 11, PPD 14)			
**log CRP**	1.43 ± 0.17	1.27 ± 0.39	0.276
**log Cortisol**	3.76 ± 1.09	3.21 ± 0.88	0.175
**log Serotonin**	8.33 ± 0.44	7.55 ± 0.44	**<0.001**

CRP, cortisol, and serotonin values represent log-transformed concentrations.

**Table 4:** Correlations between psychological variables, social support, and biomarkers among women with GDM.

Variable	Stress Score	Antepartum EPDS	Postpartum EPDS	Postpartum Social Support	CRP	Cortisol	Serotonin
Stress score	—						
Antepartum EPDS	0.494 **	—					
Postpartum EPDS	0.351 *	0.481 **	—				
Postpartum social support	0.031	0.107	0.435 **	—			
CRP	−0.079	−0.433 *	−0.473 *	−0.052	—		
Cortisol	−0.010	−0.301	−0.626 **	−0.242	0.443 *	—	
Serotonin	−0.029	−0.233	−0.616 **	−0.452 *	0.119	0.154	—

* significant at *p* < 0.05; ** significant at *p* < 0.01.

## Data Availability

The data that support the findings of this study are available from the corresponding author upon reasonable request.
